# Neuropeptide Y Reduces Social Fear in Male Mice: Involvement of Y1 and Y2 Receptors in the Dorsolateral Septum and Central Amygdala

**DOI:** 10.3390/ijms221810142

**Published:** 2021-09-20

**Authors:** Johannes Kornhuber, Iulia Zoicas

**Affiliations:** Department of Psychiatry and Psychotherapy, Friedrich-Alexander University Erlangen-Nürnberg (FAU), 91054 Erlangen, Germany; Johannes.Kornhuber@uk-erlangen.de

**Keywords:** social fear, social phobia, conditioned fear, SFC, fear expression, neuropeptide Y, BIBO3304 trifluoroacetate, BIIE0246, dorsolateral septum, central amygdala

## Abstract

Neuropeptide Y (NPY) has anxiolytic-like effects and facilitates the extinction of cued and contextual fear in rodents. We previously showed that intracerebroventricular administration of NPY reduces the expression of social fear in a mouse model of social fear conditioning (SFC) and localized these effects to the dorsolateral septum (DLS) and central amygdala (CeA). In the present study, we aimed to identify the receptor subtypes that mediate these local effects of NPY. We show that NPY (0.1 nmol/0.2 µL/side) reduced the expression of SFC-induced social fear in a brain region- and receptor-specific manner in male mice. In the DLS, NPY reduced the expression of social fear by acting on Y2 receptors but not on Y1 receptors. As such, prior administration of the Y2 receptor antagonist BIIE0246 (0.2 nmol/0.2 μL/side) but not the Y1 receptor antagonist BIBO3304 trifluoroacetate (0.2 nmol/0.2 μL/side) blocked the effects of NPY in the DLS. In the CeA, however, BIBO3304 trifluoroacetate but not BIIE0246 blocked the effects of NPY, suggesting that NPY reduced the expression of social fear by acting on Y1 receptors but not Y2 receptors within the CeA. This study suggests that at least two distinct receptor subtypes are differentially recruited in the DLS and CeA to mediate the effects of NPY on the expression of social fear.

## 1. Introduction

As the most abundant and widely distributed neuropeptide in the mammalian brain, neuropeptide Y (NPY) regulates a variety of biological and pathophysiological functions, such as blood pressure, food intake, neuroendocrine secretions, neuronal excitability and neuroplasticity [1,2,3,4,5,6]. These effects are mediated by at least five subtypes of G-protein-coupled receptors, among which the Y1, Y2, Y4 and Y5 subtypes are localized in the brain [7,8,9]. NPY and its receptors are highly expressed in brain regions involved in emotional behavior and fear-related behavior, including the amygdala, hippocampus, septum, cerebral cortex, locus coeruleus, periaqueductal gray, basal ganglia, hypothalamus and thalamus [10,11,12], suggesting a regulatory role of NPY and its receptors in these behaviors.

A considerable amount of literature demonstrates anxiolytic and fear-reducing properties of NPY that depend on the receptor subtype, brain region and dose applied. Anxiolytic-like effects were described after intracerebroventricular (i.c.v.) administration of NPY [13] and local administration of NPY into the central amygdala (CeA) [14,15], basolateral amygdala (BLA) [15], locus coeruleus [16], lateral septum [17], dorsolateral septum (DLS) [15], dentate gyrus and the CA1 region of the hippocampus [15,18], as well as after localized overexpression of NPY within the amygdala [19] or in rats with a higher innate number of NPY-positive cells in the CeA [20]. The anxiolytic-like effects observed after i.c.v. or after local administration of NPY into the CeA, lateral septum and CA1 region of the hippocampus are mediated by postsynaptic Y1 receptors [14,17,18,21], whereas the intra-dentate gyrus effects are mediated by presynaptic Y2 receptors [18]. These studies suggest a brain region- and receptor-specific modulation of anxiety-like behavior by NPY. While postsynaptic Y1 receptors were shown to mediate primarily anxiolytic-like effects [14,17,18,21], the activation of presynaptic Y2 receptors exerts predominantly anxiogenic-like effects, as demonstrated in studies using specific Y2 receptor agonists and antagonists [22,23].

Similar brain region-and receptor-specific effects of NPY have been described on social interaction, a test that simultaneously assesses social anxiety-like behavior and social motivation. As such, NPY promotes social interaction when administered into the DLS [24] and BLA [25] but does not affect social interaction when administered into the intramedial septum [24] and CeA [25]. While the effects of NPY on social interaction are mediated by Y1 receptors but not Y2 receptors in the DLS [24], both Y1 and Y2 receptors mediate its effects in the BLA [25,26]. These partly differential effects of NPY on anxiety (i.e., non-social anxiety) and social anxiety suggest that distinct brain regions and receptor subtypes mediate the effects of NPY on social versus non-social anxiety.

Several studies using fear conditioning paradigms have revealed that NPY is also a strong suppressor of fear. Accordingly, i.c.v.-administered NPY impairs the acquisition of cued and contextual fear by acting on Y1 receptors [27,28,29], impairs the retention and retrieval of cued fear by acting on Y1 and Y2 receptors [30,31,32] and facilitates the extinction of cued and contextual fear possibly by simultaneously acting on Y1 and Y2 receptors and also on Y4 receptors [29,30,33,34,35]. The effects of NPY on the expression of cued fear also seem to be brain region-dependent since NPY inhibits the expression of cued fear when administered into the BLA but not when administered into the medial amygdala (MeA) [30,31] via a Y1 receptor-independent mechanism [31].

These anxiolytic-like, prosocial and fear-reducing properties of NPY suggest its potential benefit in disorders associated with social anxiety and fear. Indeed, in a model of social fear conditioning (SFC), we showed that i.c.v. administration of NPY reduced the expression of social fear by simultaneously acting on Y1 and Y2 receptors [36]. Moreover, i.c.v. administration of NPY also reduced the expression of antidepressant-resistant social fear [37], further supporting its potential benefit in disorders associated with social anxiety and fear. The effects of NPY on the expression of social fear are also brain region-dependent and could be localized to the DLS and CeA. As such, NPY reduced the expression of social fear when administered into the DLS and CeA but not when administered into the dorsal hippocampus, MeA and BLA [15].

As a follow-up study, we aimed to identify the receptor subtypes that mediate these local effects of NPY on the expression of social fear given that receptor-specific effects of NPY have been reported on other types of behavior, including anxiety-like behavior, social behavior and fear-related behavior. As the pharmacological inactivation of either Y1 or Y2 receptors reduced the i.c.v. effects of NPY on the expression of social fear, and simultaneous inactivation of these receptors completely blocked the i.c.v. effects of NPY [36], we administered specific Y1 and Y2 receptor antagonists before NPY into the DLS and CeA prior to social fear extinction.

## 2. Results

**Figure 1:**
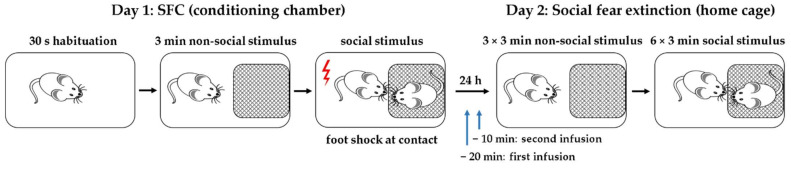
Schematic representation of the social fear conditioning (SFC) paradigm. On day 1, during SFC, the mice were placed in the conditioning chamber and, after a 30 s habituation period, an empty wire mesh cage was placed as a non-social stimulus near one of the short walls. After 3 min, the non-social stimulus was replaced by an identical cage containing an unfamiliar mouse, i.e., social stimulus. Mice were given a mild electric foot shock each time they investigated the social stimulus. On day 2, during social fear extinction, the mice were exposed in their home cages to three non-social stimuli, followed by exposure to six unfamiliar social stimuli. Twenty min before social fear extinction, the mice received the first infusion (vehicle, BIBO3304 trifluoroacetate or BIIE0246). Ten min later, the mice were infused again with either vehicle or NPY.

To investigate whether the intra-DLS and intra-CeA administration of NPY reduces the expression of social fear via Y1 and/or Y2 receptors, separate groups of conditioned mice were administered vehicle (Veh; 0.2 µL/side), BIBO3304 trifluoroacetate (BIBO; Y1 receptor antagonist; 0.2 nmol/0.2 μL/side) or BIIE0246 (BIIE; Y2 receptor antagonist; 0.2 nmol/0.2 μL/side) into these brain regions 20 min before social fear extinction on Day 2. After 10 min, mice were infused again with either Veh (0.2 μL/side) or NPY (0.1 nmol/0.2 μL/side). On Day 1, during SFC, mice received mild electric foot shocks each time they investigated an unfamiliar mouse to induce social fear. On Day 2, during social fear extinction, the time that the mice spent investigating three empty cages (i.e., non-social investigation) and six unfamiliar mice enclosed in wire mesh cages (i.e., social investigation) was assessed as a read-out of non-social and social fear, respectively (Figure 1).

### 2.1. NPY Reduces the Expression of Social Fear by Acting on Y2 Receptors in the DLS

**Figure 2:**
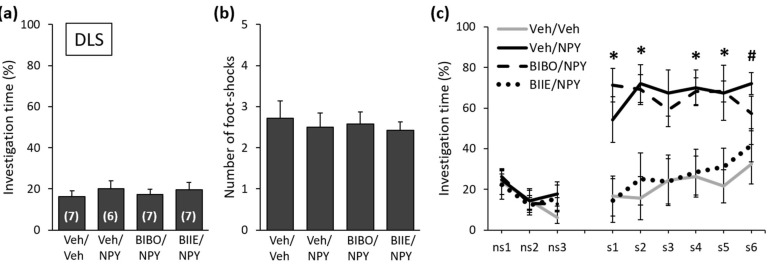
NPY reduces the expression of social fear by acting on Y2 receptors in the dorsolateral septum (DLS). (**a**) Preconditioning investigation of the non-social stimulus (empty cage) during SFC on Day 1. (**b**) Number of foot shocks received during SFC. (**c**) Investigation of the non-social (ns1–ns3) and social (cages with mice; s1–s6) stimuli during social fear extinction on Day 2. Conditioned mice were bilaterally infused with either vehicle (Veh; 0.2 µL/side), BIBO3304 trifluoroacetate (BIBO; Y1 receptor antagonist; 0.2 nmol/0.2 µL/side) or BIIE0246 (BIIE; Y2 receptor antagonist; 0.2 nmol/0.2 µL/side) into the DLS 20 min before social fear extinction on Day 2. After 10 min, mice were infused again with Veh (0.2 µL/side) or NPY (0.1 nmol/0.2 µL/side). NPY reduced the expression of social fear; this effect was blocked by BIIE but not by BIBO (treatment effect: F (3,23) = 12.195; *p* < 0.001; stimulus x treatment effect: F (24,184) = 2.829; *p* < 0.001). Data represent the means ± SEM, and numbers in parentheses indicate group sizes. *p* < 0.05 * Veh/NPY and BIBO/NPY vs. Veh/Veh, and Veh/NPY vs. BIIE/NPY; #Veh/NPY vs. Veh/Veh.

During SFC on Day 1, the mice showed similar levels of investigation of the non-social stimulus (empty cage), reflecting similar preconditioning non-social anxiety between the groups (Figure 2a; F (3,23) = 0.326; *p* = 0.807). All mice received a similar number of foot shocks during SFC, reflecting similar levels of distress during conditioning and similar social fear learning between the groups (Figure 2b; F (3,23) = 0.145; *p* = 0.932). During social fear extinction on Day 2, all mice showed similar levels of investigation of the non-social stimuli (three empty cages; ns1–ns3), indicating that the treatment did not alter non-social fear (Figure 2c). While the Veh/NPY-treated mice showed increased social investigation compared with the Veh/Veh-treated mice, which indicated that NPY reduced the expression of social fear when administered into the DLS, BIIE0246 but not BIBO3304 trifluoroacetate blocked these effects of NPY (treatment effect: F (3,23) = 12.195; *p* < 0.001; stimulus x treatment effect: F (24,184) = 2.829; *p* < 0.001). In more detail, the BIBO/NPY- and Veh/NPY-treated mice showed increased social investigation compared with the Veh/Veh-treated mice, indicating that the Y1 receptor antagonist BIBO3304 trifluoroacetate did not block the effects of NPY on the expression of social fear. The BIIE/NPY-treated mice, however, showed a similarly low level of social investigation compared with the Veh/Veh-treated mice, indicating that the Y2 receptor antagonist BIIE0246 blocked the effects of NPY on the expression of social fear. These results demonstrate that NPY reduces the expression of social fear by acting on Y2 but not Y1 receptors in the DLS.

### 2.2. NPY Reduces the Expression of Social Fear by Acting on Y1 Receptors in the CeA

**Figure 3:**
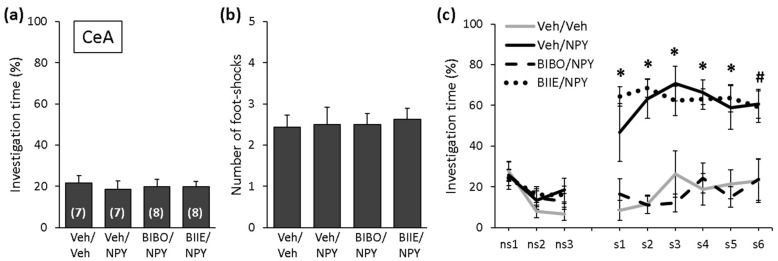
NPY reduces the expression of social fear by acting on Y1 receptors in the central amygdala (CeA). (**a**) Preconditioning investigation of the non-social stimulus (empty cage) during SFC on Day 1. (**b**) Number of foot shocks received during SFC. (**c**) Investigation of the non-social (ns1–ns3) and social (cages with mice; s1–s6) stimuli during social fear extinction on Day 2. Conditioned mice were bilaterally infused with either vehicle (Veh; 0.2 µL/side), BIBO3304 trifluoroacetate (BIBO; Y1 receptor antagonist; 0.2 nmol/0.2 µL/side) or BIIE0246 (BIIE; Y2 receptor antagonist; 0.2 nmol/0.2 µL/side) into the CeA 20 min before social fear extinction on Day 2. After 10 min, mice were infused again with Veh (0.2 µL/side) or NPY (0.1 nmol/0.2 µL/side). NPY reduced the expression of social fear; this effect was blocked by BIBO but not by BIIE (treatment effect: F (3,26) = 17.896; *p* < 0.001; stimulus x treatment effect: F (24,208) = 5.520; *p* < 0.001). Data represent the means ± SEM, and numbers in parentheses indicate group sizes. *p* < 0.05 * Veh/NPY and BIIE/NPY vs. Veh/Veh, and Veh/NPY vs. BIBO/NPY; # Veh/NPY vs. BIBO/NPY.

During SFC on Day 1, mice showed similar levels of investigation of the non-social stimulus (empty cage), reflecting similar preconditioning non-social anxiety between the groups (Figure 3a; F (3,26) = 0.124; *p* = 0.945). All mice received a similar number of foot shocks during SFC, reflecting similar levels of distress during conditioning and similar social fear learning between the groups (Figure 3b; F (3,26) = 0.069; *p* = 0.976). During social fear extinction on Day 2, all mice showed similar levels of investigation of the non-social stimuli (three empty cages; ns1–ns3), indicating that the treatment did not alter non-social fear (Figure 3c). While the Veh/NPY-treated mice showed increased social investigation compared with the Veh/Veh-treated mice, which indicated that NPY reduced the expression of social fear when administered into the CeA, BIBO3304 trifluoroacetate but not BIIE0246 blocked these effects of NPY (treatment effect: F (3,26) = 17.896; *p* < 0.001; stimulus x treatment effect: F (24,208) = 5.520; *p* < 0.001). In more detail, the BIIE/NPY-and Veh/NPY-treated mice showed an increased social investigation compared with the Veh/Veh-treated mice, indicating that the Y2 receptor antagonist BIIE0246 did not block the effects of NPY on the expression of social fear. The BIBO/NPY-treated mice, however, showed a similarly low level of social investigation compared with the Veh/Veh-treated mice, indicating that the Y1 receptor antagonist BIBO3304 trifluoroacetate blocked the effects of NPY on the expression of social fear. These results demonstrate that NPY reduces the expression of social fear by acting on Y1 but not Y2 receptors in the CeA.

## 3. Discussion

The present study demonstrates for the first time that NPY reduces the expression of SFC-induced social fear in a brain region-and receptor-specific manner in male mice. In more detail, we showed that when administered into the DLS, NPY reduced the expression of social fear by acting on Y2 receptors but not on Y1 receptors. When administered into the CeA, however, NPY reduced the expression of social fear by acting on Y1 receptors but not on Y2 receptors. These results suggest that at least two distinct receptor subtypes are differentially recruited in the DLS and CeA to mediate the effects of NPY on the expression of social fear.

In previous studies, we have shown that i.c.v. administration of NPY reduced the expression of SFC-induced social fear via simultaneous activation of Y1 and Y2 receptors [36]. While both the Y1 receptor antagonist BIBO3304 trifluoroacetate and the Y2 receptor antagonist BIIE0246 partly blocked the effects of NPY on social fear expression, only the combination of these antagonists completely blocked the effects of NPY. I.c.v. administration of NPY also reduced the expression of antidepressant-resistant social fear in mice lacking the lysosomal glycoprotein acid sphingomyelinase (ASM) [37]. In these ASM-/- mice, antidepressants such as paroxetine and amitriptyline failed to reverse SFC-induced social fear, although they were successful in reversing social fear in wild-type ASM+/+ mice [37} and in CD1 mice [38]. This further supports the potential benefit of NPY in disorders associated with social anxiety and fear and its potential as an alternative pharmacotherapy for social anxiety disorder patients who fail to respond to antidepressant treatments [39]. The effects of NPY on the expression of social fear seem to be brain region-dependent since NPY reduced the expression of social fear when administered into the DLS and CeA but not when administered into the dorsal hippocampus, MeA and BLA [15]. In this study, we confirmed and extended these findings by identifying the receptor subtypes that mediate the effects of NPY in the DLS and CeA.

The lateral septum plays a critical role in regulating emotional behaviors, social behaviors and fear-related behaviors [40] and expresses both presynaptic Y1 and postsynaptic Y2 receptors [41,42]. When administered into the DLS, NPY was shown to exert anxiolytic-like effects [15], to stimulate social interaction by acting on Y1 receptors but not on Y2 receptors [24] and to reduce the expression of social fear [15]. Although Kask et al. [24] showed that NPY stimulated social interaction when administered into the DLS, it seems unlikely that DLS-administered NPY reduced the expression of social fear simply by stimulating social interaction. First, different receptor subtypes mediated the effects of NPY on social interaction and on social fear expression. As such, NPY stimulated social interaction by acting on Y1 receptors but not on Y2 receptors [24], whereas the effects on social fear expression were mediated by Y2 receptors and not by Y1 receptors (Figure 2c). Second, NPY increased social investigation only in socially fear conditioned SFC+ mice but not in unconditioned control SFC− mice when administered into the DLS and CeA [15], suggesting that NPY increases social investigation only in individuals with low or impaired sociability. This effect was also observed after i.c.v. administration of NPY [36,37] and resembles the effects of other neuropeptides, such as oxytocin or neuropeptide S, which were also shown to reduce social fear in SFC+ mice without further increasing social investigation in SFC− mice [43,44]. Given that the anxiolytic-like effects of NPY in the lateral septum were mediated by Y1 receptors [17], it is possible that NPY reduced the expression of social fear exclusively by acting on Y2 receptors or by simultaneously acting on Y1 and Y2 receptors. While Y2 receptors mediate the effects of NPY on social fear extinction, it cannot be excluded that the anxiolytic-like effects exerted via Y1 receptors contributed to the reduced expression of social fear. By reducing anxiety, NPY might enable the mice to approach the social stimuli faster and therefore lead to a faster extinction of social fear. Interestingly, the DLS seems to be an important brain region for the development and expression of social fear, and several neuropeptide classes can modulate social fear at the level of the DLS. For example, local administration of neuropeptides with anxiolytic and prosocial properties such as oxytocin and NPY in the DLS reduced the expression of social fear [43,45] (Figure 2c). Moreover, social fear increased oxytocin receptor binding and impaired the release of oxytocin within the DLS [43], which were alterations that normalized after successful extinction of social fear. By using c-Fos immunohistochemistry, Menon et al. [45] demonstrated reduced neuronal activation within the lateral septum in lactating mice that showed reduced expression of SFC-induced social fear, further demonstrating the involvement of the lateral septum in the development and expression of social fear. Preliminary studies have suggested that social fear also alters the expression of NPY system-related genes. For example, we observed increased *Npyr2* gene expression in the lateral septum of SFC+ mice, suggesting the involvement of this receptor in social fear and supporting our results showing that NPY reduced the expression of social fear by activating Y2 receptors within the DLS.

The amygdala is the central component of the fear circuitry and is involved in the perception, learning and expression of fear [46]. Within the amygdala, the CeA constitutes the output relay for the functional consequences of amygdala activation by fearful stimuli and, together with the BLA, coordinates the behavioral and physiological correlates of fear expression [46,47]. When administered into the CeA, NPY was shown to exert anxiolytic-like effects by acting on Y1 receptors [14,15] and to reduce the expression of social fear by acting on Y1 receptors but not on Y2 receptors [15] (Figure 3c). This suggests that the effects of NPY on social fear expression might be at least partly mediated by its anxiolytic-like effects. As NPY did not promote social interaction when administered into the CeA [15,25], it seems unlikely that CeA-administered NPY reduced the expression of social fear by stimulating social interaction. In a study employing viral vector-mediated overexpression of the Y2 selective agonist NPY3-36, Verma et al. [34] demonstrated that NPY promoted cued fear extinction by reducing the expression of cued fear via activation of Y2 receptors within the CeA. This suggests that NPY reduces the expression of both social and cued fear at the level of CeA and that distinct receptor subtypes are recruited to mediate these effects depending on the valence of the stimulus. As such, activation of postsynaptic Y2 receptors within the CeA reduces the expression of cued fear and thereby promotes the extinction of cued fear but does not affect the expression and extinction of social fear. Activation of presynaptic Y1 receptors, on the other hand, reduces the expression of social fear and thereby promotes social fear extinction.

The different contributions of Y1 and Y2 receptors in the regulation of conditioned social fear at the level of the DLS and CeA are unlikely explained by a different anatomical localization of the receptors, as both the presynaptic Y1 and postsynaptic Y2 receptors are expressed in the DLS and CeA. Quantitative receptor autoradiography studies revealed moderate levels of Y1 receptor expression and high levels of Y2 receptor expression in both the DLS and the CeA [41,42]. 

Although we were able to identify the receptors mediating the effects of NPY on the expression of social fear at the level of the DLS and CeA, a simultaneous activation of Y1 and Y2 receptors was not necessary in any of these brain regions. Given that activation of both Y1 and Y2 receptors was necessary to mediate the i.c.v. effects of NPY on the expression of social fear [15], it is possible that the effects observed after i.c.v. administration of NPY were mediated simultaneously by postsynaptic Y2 receptors within the DLS and by presynaptic Y1 receptors within the CeA. The question arises on whether additional brain regions might also be recruited and whether the activation of both the Y1 and Y2 receptors in these brain regions might be necessary to mediate the effects of NPY on the expression of social fear. Brain regions such as the dorsal hippocampus, MeA and BLA do not seem to mediate the effects of NPY on social fear [15]. However, the involvement of other brain regions expressing Y1 and Y2 receptors and known to be associated with social behavior and fear-related behavior, such as the prefrontal cortex and the bed nucleus of the stria terminalis [48,49], has not been investigated so far.

Taken together, we have shown that when administered into the DLS, NPY reduced the expression of social fear by acting on Y2 receptors but not on Y1 receptors. When administered into the CeA, however, NPY reduced the expression of social fear by acting on Y1 receptors but not on Y2 receptors. These results suggest that at least two distinct receptor subtypes are differentially recruited in the DLS and CeA to mediate the effects of NPY on the expression of social fear.

## 4. Materials and Methods

### 4.1. Animals

Male CD1 mice (Charles River, Sulzfeld, Germany, 8 weeks of age, 32–36 g) were individually housed after arrival and remained single-housed throughout the experiments. Age- and weight-matched male CD1 mice were used as social stimuli. Mice were kept under standard laboratory conditions (12:12 light/dark cycle, lights on at 07:00 h, 22 °C, 60% humidity, food and water ad libitum). Experiments were performed during the light phase between 09:00 and 14:00. All efforts were made to minimize animal suffering and to reduce the number of animals used.

### 4.2. Stereotaxic Cannula Implantation

Implantation of the guide cannula (23G, 8 mm length; Injecta GmbH, Klingenthal, Germany) for bilateral infusions was performed one week after arrival under ketamine-xylazine anesthesia (intraperitoneal injection of 120 mg/kg Ketavet^®^ and 16 mg/kg Rompun^®^, respectively) as previously described [15,50], 1 mm above the DLS (AP + 0.3 mm, L ± 0.5 mm, D + 1.6 mm) or CeA (AP-1.2 mm, L ± 2.8 mm, D + 3.8 mm). After surgery, mice were handled for 5 days before experiments started.

### 4.3. Intracerebral Infusions

Mice received bilateral DLS or CeA infusions of either vehicle (Veh; distilled H_2_O; 0.2 µL/side), porcine NPY (0.1 nmol/0.2 µL/side; PeptaNova, Sandhausen, Germany), Y1 receptor antagonist BIBO3304 trifluoroacetate (BIBO; *N*-[(1*R*)-1-[[[[4-[[(Aminocarbonyl)amino]methyl]phenyl]methyl]amino]carbonyl]-4-[(aminoiminomethyl)amino]butyl]-α-phenyl-benzeneacetamide ditrifluoroacetate; 0.2 nmol/0.2 μL/side; Tocris Bioscience, Bristol, UK) or Y2 receptor antagonist BIIE0246 (BIIE; *N*-[(1*S*)-4-[(Aminoiminomethyl)amino]-1-[[[2-(3,5-dioxo-1,2-diphenyl-1,2,4-triazolidin-4-yl)ethyl]amino]carbonyl]butyl]-1-[2-[4-(6,11-dihydro-6-oxo-5*H*-dibenz[*b*,*e*]azepin-11-yl)-1-piperazinyl]-2-oxoethyl]-cyclopentaneacetamide*;* 0.2 nmol/0.2 μL/side; Tocris Bioscience, Bristol, UK) via an infusion cannula (27G, 9 mm length) inserted into the guide cannula and connected via polyethylene tubing to a Hamilton syringe. The infusion system was left in place for 30 s following the infusion to allow diffusion of the solution.

The correct infusion site was histologically verified. Brains were removed, snap-frozen in isopentane (2-Methylbutane; Sigma-Aldrich, Darmstadt, Germany) and dry ice and cut in coronal brain sections through the guide cannula probe tracts. Accordingly, 3 DLS cannulas were not correctly implanted, and these mice were excluded from the study. NPY, BIBO3304 trifluoroacetate and BIIE0246 doses and timing of administration were selected based on previous studies [15,28,50].

### 4.4. Social Fear Conditioning (SFC) Paradigm

To induce social fear, mice were conditioned during SFC, and social investigation was assessed during social fear extinction as a read-out of social fear.

*SFC.* SFC was performed with a computerized fear conditioning system (TSE System GmbH, Bad Homburg, Germany) as previously described [36,37,38,43,44,51,52]; see [53] for a schematic representation of the SFC paradigm. The mice were placed in the conditioning chamber (45 × 22 × 40 cm), and after a 30-s habituation period, an empty wire mesh cage (7 × 7 × 6 cm) was placed as a non-social stimulus near one of the short walls. After 3 min, the non-social stimulus was replaced by an identical cage containing an unfamiliar male mouse, i.e., social stimulus. Mice were given a 1-s mild electric foot shock (0.7 mA) each time they investigated, i.e., made direct contact with the social stimulus. The mice received between 2 and 4 foot shocks, with a variable inter-shock interval, depending on when direct social contact was made. The number of foot shocks was assessed as a measure of distress and social fear learning. The mice were returned to their home cage when no further social contact was made for 2 min (average duration of SFC approximately 10 min). All mice investigated the social stimulus and could be conditioned. The time mice spent investigating the non-social stimulus, as a preconditioning measure of non-social anxiety, was analyzed.

*Social fear extinction.* One day after SFC, the mice were exposed in their home cage to three non-social stimuli, i.e., empty cages identical to the cage used during SFC, to assess non-social investigation as a parameter of non-social fear. The mice were then exposed to six unfamiliar social stimuli, i.e., mice enclosed in wire mesh cages, to assess social investigation as a parameter of social fear. Each stimulus was placed near a short wall of the home cage and presented for 3 min, with a 3 min inter-exposure interval. The test was recorded and analyzed using JWatcher (V 1.0, Macquarie University, Sydney, Australia, and UCLA, Los Angeles, CA, USA). Non-social investigation was defined as direct sniffing of the empty cage, whereas social investigation was defined as direct sniffing of the cage and/or the social stimulus inside the cage.

### 4.5. Statistical Analysis

For the statistical analysis, SPSS (Version 24, SPSS Inc., Chicago, IL, USA) was used. Data were analyzed by one-way ANOVA or two-way ANOVA for repeated measures, followed by Bonferroni’s post hoc analysis whenever appropriate. Statistical significance was set at *p* < 0.05.

## Data Availability

The datasets generated during the current study are available from the corresponding author on request.

## References

[1] Stanley B.G., Leibowitz S.F. (1984). Neuropeptide Y: Stimulation of feeding and drinking by injection into the paraventricular nucleus. Life Sci..

[2] Colmers W.F., Bleakman D. (1994). Effects of neuropeptide Y on the electrical properties of neurons. Trends Neurosci..

[3] Vezzani A., Sperk G., Colmers W.F. (1999). Neuropeptide Y: Emerging evidence for a functional role in seizure modulation. Trends Neurosci..

[4] Michalkiewicz M., Michalkiewicz T., Kreulen D.L., McDougall S. (2001). Increased blood pressure responses in neuropeptide Y transgenic rats. Am. J. Physiol. Integr. Comp. Physiol..

[5] Magni P. (2003). Hormonal Control of the Neuropeptide Y System. Curr. Protein Pept. Sci..

[6] Hökfelt T., Stanic D., Sanford S.D., Gatlin J.C., Nilsson I., Paratcha G., Ledda F., Fetissov S., Lindfors C., Herzog H. (2008). NPY and its involvement in axon guidance, neurogenesis, and feeding. Nutrition.

[7] Dumont Y., Fournier A., St-Pierre S., Quirion R. (1993). Comparative characterization and autoradiographic distribution of neuropeptide Y receptor subtypes in the rat brain. J. Neurosci..

[8] Dumont Y., Fournier A., St-Pierre S. (1996). Autoradiographic distribution of [125I]Leu31,Pro34]PYY and [125I]PYY3-36 binding sites in the rat brain evaluated with two newly developed Y1 and Y2 receptor radioligands. Synapse.

[9] Parker R.M.C., Herzog H. (1999). Regional distribution of Y-receptor subtype mRNAs in rat brain. Eur. J. Neurosci..

[10] Chang R.S., Lotti V.J., Chen T.-B., Cerino D.J., Kling P.J. (1985). Neuropeptide Y (NPY) binding sites in rat brain labeled with 125I-Bolton-Hunter NPY: Comparative potencies of various polypeptides on brain NPY binding and biological responses in the rat vas deferens. Life Sci..

[11] Quidt M.E., Emson P.C. (1986). Distribution of neuropeptide Y-like immunoreactivity in the rat central nervous system-II. Immunohistochemical analysis. Neuroscience.

[12] Lynch D.R., Walker M.W., Miller R.J., Snyder S.H. (1989). Neuropeptide Y receptor binding sites in rat brain: Differential autoradiographic localizations with 125I-peptide YY and 125I-neuropeptide Y imply receptor heterogeneity. J. Neurosci..

[13] Heilig M., Söderpalm B., Engel J.A., Widerlöv E. (1989). Centrally administered neuropeptide Y (NPY) produces anxiolytic-like effects in animal anxiety models. Psychopharmacology.

[14] Heilig M., McLeod S., Brot M.D., Heinrichs S.C., Menzaghi F., Koob G.F., Britton K.T. (1993). Anxiolytic-Like Action of Neuropeptide Y: Mediation by Y1 Receptors in Amygdala, and Dissociation from Food Intake Effects. Neuropsychopharmacology.

[15] Kornhuber J., Zoicas I. (2021). Brain Region-Dependent Effects of Neuropeptide Y on Conditioned Social Fear and Anxiety-Like Behavior in Male Mice. Int. J. Mol. Sci..

[16] Kask A., Rägo L., Harro J. (1998). Anxiolytic-like effect of neuropeptide Y (NPY) and NPY13–36 microinjected into vicinity of locus coeruleus in rats. Brain Res..

[17] Trent N.L., Menard J.L. (2011). Infusions of neuropeptide Y into the lateral septum reduce anxiety-related behaviors in the rat. Pharmacol. Biochem. Behav..

[18] Śmiałowska M., Wierońska J.M., Domin H., Zięba B. (2007). The Effect of Intrahippocampal Injection of Group II and III Metobotropic Glutamate Receptor Agonists on Anxiety; the Role of Neuropeptide, Y. Neuropsychopharmacology.

[19] Thorsell A., Repunte-Canonigo V., O’Dell L.E., Chen S.A., King A.R., Lekic D., Koob G.F., Sanna P.P. (2007). Viral vector-induced amygdala NPY overexpression reverses increased alcohol intake caused by repeated deprivations in Wistar rats. Brain.

[20] Sharko A.C., Kaigler K.F., Fadel J.R., Wilson M.A. (2016). Ethanol-induced anxiolysis and neuronal activation in the amygdala and bed nucleus of the stria terminalis. Alcohol.

[21] Sørensen G., Lindberg C., Wörtwein G., Bolwig T.G., Woldbye D.P. (2004). Differential roles for neuropeptide Y Y1 and Y5 receptors in anxiety and sedation. J. Neurosci. Res..

[22] Nakajima M., Inui A., Asakawa A., Momose K., Ueno N., Teranishi A., Baba S., Kasuga M. (1998). Neuropeptide Y Produces Anxiety Via Y2-Type Receptors. Peptides.

[23] Bacchi F., Mathé A.A., Jiménez P., Stasi L., Arban R., Gerrard P., Caberlotto L. (2006). Anxiolytic-like effect of the selective Neuropeptide Y Y2 receptor antagonist BIIE0246 in the elevated plus-maze. Peptides.

[24] Kask A., Nguyen H.P., Pabst R., Von Hörsten S. (2001). Neuropeptide Y Y1 receptor-mediated anxiolysis in the dorsocaudal lateral septum: Functional antagonism of corticotropin-releasing hormone-induced anxiety. Neuroscience.

[25] Sajdyk T.J., Vandergriff M., Gehlert D.R. (1999). Amygdalar neuropeptide Y Y1 receptors mediate the anxiolytic-like actions of neuropeptide Y in the social interaction test. Eur. J. Pharmacol..

[26] Sajdyk T.J., Schober D.A., Smiley D.L., Gehlert D.R. (2002). Neuropeptide Y-Y2 receptors mediate anxiety in the amygdala. Pharmacol. Biochem. Behav..

[27] Broqua P., Wettstein J., Rocher M., Gauthier-Martin B., Junien J. (1995). Behavioral effects of neuropeptide Y receptor agonists in the elevated plus-maze and fear-potentiated startle procedures. Behav. Pharmacol..

[28] Karlsson R.-M., Holmes A., Heilig M., Crawley J.N. (2005). Anxiolytic-like actions of centrally-administered neuropeptide Y, but not galanin, in C57BL/6J mice. Pharmacol. Biochem. Behav..

[29] Lach G., de Lima T.C. (2013). Role of NPY Y1 receptor on acquisition, consolidation and extinction on contextual fear conditioning: Dissociation between anxiety, locomotion and non-emotional memory behavior. Neurobiol. Learn. Mem..

[30] Gutman A.R., Yang Y., Ressler K.J., Davis M. (2008). The Role of Neuropeptide Y in the Expression and Extinction of Fear-Potentiated Startle. J. Neurosci..

[31] Fendt M., Bürki H., Imobersteg S., Lingenhöhl K., McAllister K.H., Orain D., Uzunov D.P., Chaperon F. (2009). Fear-reducing effects of intra-amygdala neuropeptide Y infusion in animal models of conditioned fear: An NPY Y1 receptor independent effect. Psychopharmacology.

[32] Pickens C., Adams-Deutsch T., Nair S., Navarre B., Heilig M., Shaham Y. (2009). Effect of pharmacological manipulations of neuropeptide Y and corticotropin-releasing factor neurotransmission on incubation of conditioned fear. Neuroscience.

[33] Verma D., Tasan R.O., Herzog H., Sperk G. (2012). NPY controls fear conditioning and fear extinction by combined action on Y₁ and Y₂ receptors. Br. J. Pharmacol..

[34] Verma D., Wood J., Lach G., Mietzsch M., Weger S., Heilbronn R., Herzog H., Bonaventure P., Sperk G., Tasan R. (2015). NPY Y2 receptors in the central amygdala reduce cued but not contextual fear. Neuropharmacology.

[35] Verma D., Hörmer B., Bellmann-Sickert K., Thieme V., Beck-Sickinger A.G., Herzog H., Sperk G., Tasan R.O. (2016). Pancreatic polypeptide and its central Y 4 receptors are essential for cued fear extinction and permanent suppression of fear. Br. J. Pharmacol..

[36] Kornhuber J., Zoicas I. (2019). Neuropeptide Y reduces expression of social fear via simultaneous activation of Y1 and Y2 receptors. J. Psychopharmacol..

[37] Kornhuber J., Zoicas I. (2020). Neuropeptide Y as Alternative Pharmacotherapy for Antidepressant-Resistant Social Fear. Int. J. Mol. Sci..

[38] Toth I., Neumann I.D., Slattery D.A. (2012). Social Fear Conditioning: A Novel and Specific Animal Model to Study Social Anxiety Disorder. Neuropsychopharmacology.

[39] Blanco C., Antia S.X., Liebowitz M.R. (2002). Pharmacotherapy of social anxiety disorder. Biol. Psychiatry.

[40] Sheehan T.P., Chambers R., Russell D.S. (2004). Regulation of affect by the lateral septum: Implications for neuropsychiatry. Brain Res. Rev..

[41] Tasan R., Verma D., Wood J., Lach G., Hörmer B., de Lima T.C.M., Herzog H., Sperk G. (2016). The role of Neuropeptide Y in fear conditioning and extinction. Neuropeptides.

[42] Dumont Y., Jacques D., Bouchard P., Quirion R. (1998). Species differences in the expression and distribution of the neuropeptide Y Y1, Y2, Y4, and Y5 receptors in rodents, guinea pig, and primates brains. J. Comp. Neurol..

[43] Zoicas I., Slattery A.D., Neumann I.D. (2014). Brain Oxytocin in Social Fear Conditioning and Its Extinction: Involvement of the Lateral Septum. Neuropsychopharmacology.

[44] Zoicas I., Menon R., Neumann I.D. (2016). Neuropeptide S reduces fear and avoidance of con-specifics induced by social fear conditioning and social defeat, respectively. Neuropharmacology.

[45] Menon R., Grund T., Zoicas I., Althammer F., Fiedler D., Biermeier V., Bosch O.J., Hiraoka Y., Nishimori K., Eliava M. (2018). Oxytocin Signaling in the Lateral Septum Prevents Social Fear during Lactation. Curr. Biol..

[46] LeDoux J.E. (2007). The amygdala. Curr. Biol..

[47] Fanselow M.S., LeDoux E.J. (1999). Why We Think Plasticity Underlying Pavlovian Fear Conditioning Occurs in the Basolateral Amygdala. Neuron.

[48] Wang J., Tian Y., Zeng L.-H., Xu H. (2020). Prefrontal Disinhibition in Social Fear: A Vital Action of Somatostatin Interneurons. Front. Cell. Neurosci..

[49] Lebow M.A., Chen A. (2016). Overshadowed by the amygdala: The bed nucleus of the stria terminalis emerges as key to psychiatric disorders. Mol. Psychiatry.

[50] Kornhuber J., Zoicas I. (2020). Neuropeptide Y prolongs non-social memory in a brain region- and receptor-specific way in male mice. Neuropharmacology.

[51] Kornhuber J., Zoicas I. (2020). Social fear memory requires two stages of protein synthesis in mice. Int. J. Mol. Sci..

[52] Toth I., Neumann I.D., Slattery D.A. (2013). Social Fear Conditioning as an Animal Model of Social Anxiety Disorder. Curr. Protoc. Neurosci..

[53] Toth I., Neumann I.D. (2013). Animal models of social avoidance and social fear. Cell Tissue Res..

